# Hsa_circ_0005986 inhibits carcinogenesis by acting as a miR-129-5p sponge and is used as a novel biomarker for hepatocellular carcinoma

**DOI:** 10.18632/oncotarget.16709

**Published:** 2017-03-30

**Authors:** Liyun Fu, Qingqing Chen, Ting Yao, Tianwen Li, Sheng Ying, Yaoren Hu, Junming Guo

**Affiliations:** ^1^ Department of Hepatology, Ningbo No. 2 Hospital, and the Affiliated Hospital, Medical School of Ningbo University, Ningbo 315010, China; ^2^ Department of Biochemistry and Molecular Biology, Zhejiang Key Laboratory of Pathophysiology, Medical School of Ningbo University, Ningbo 315211, China

**Keywords:** circular RNA, hsa_circ_0005986, hepatocellular carcinoma, biomarker, miR-129-5p

## Abstract

Circular RNAs (circRNAs), a class of long-time-ignored noncoding RNA, have been revealed as multifunctional RNAs in recent years. However, the diagnostic values and the mechanism of most circRNAs in hepatocellular carcinoma (HCC) remain unknown. In this study, we revealed that the expression level of hsa_circ_0005986 in HCC was significantly lower than that in adjacent non-tumorous tissues (*P* < 0.001). Its levels in HCC cell lines, HepG2, SMMC7721, Huh7, MHCC97L, MHCC97H, and HCCLM3 were significantly lower than those in human normal hepatic cell line L02 (*P* < 0.001). In addition, the low expression level of hsa_circ_0005986 was correlated with chronic hepatitis B family history (*P* = 0.001), tumor diameters (*P* < 0.001), microvascular invasion (*P* = 0.026), and Barcelona Clinic Liver Cancer (BCLC) stage (*P* < 0.001). Further experiments demonstrated that both hsa_circ_0005986 and *Notch1*mRNA were targets of miR-129-5p, and that hsa_circ_0005986 downregulation liberated miR-129-5p and decreased the expression level of *Notch1*mRNA. More importantly, hsa_circ_0005986 downregulation accelerated cell proliferation by promoting the G_0_/G_1_ to S phase transition. We conclude that hsa_circ_0005986 function as microRNA sponge in tumorigenesis and can be used as a novel biomarker for HCC.

## INTRODUCTION

Hepatocellular carcinoma (HCC) is the most common type of primary liver cancer [1, 2]. HCC ranks the fourth in the frequency of cancer in men [3]. Patients diagnosed at the early stages of HCC can be successfully cured; however, there is deficiency of effective methods for patients diagnosed at the advanced stages [4]. Therefore, to reduce the economic and social burden, screening methods for detecting HCC at an earlier stage are highly demanded.

In recent years, circular RNAs (circRNAs), a class of endogenous RNAs have been found to widely exist in mammalian cells, have been receiving extensive attention by researchers [5–7]. By interacting with microRNAs (miRNAs) or other molecules, circRNAs regulate gene expression at the transcriptional or post-transcriptional level [8]. Due to a lack of 3′ and 5′ ends and resistance to RNases, circRNAs might be used as potential biomarkers and treatment targets for cancers [6, 9]. Recently, researchers have reported that circRNAs are involved in carcinogenesis of several types of cancers, such as gastric cancer, colorectal cancer, ovarian cancer and HCC [6, 9–12]. Harboring miRNA response elements (MREs), some circRNAs act as competing endogenous RNAs (ceRNAs) to play their biological roles, especially in cancer. For examples, circHIPK3 regulates cell growth by sponging multiple miRNAs [13]; circITCH plays an inhibitory role in esophageal squamous cell carcinoma (ESCC) by suppressing the Wnt/β-catenin pathway through interacting with multiple miRNAs [14]; Cdr1 antisense locus (CDR1as) acts as an oncogene partly through targeting miR-7 in HCC [15]. However, the function of most circRNAs in HCC and their clinical significance remain unknown.

Hsa_circ_0005986 is one of the upregulated circRNAs, with fold change 5.841, in our microarray screen detected by comparing HCC tissues with adjacent non-tumorous tissues (GEO No. 94508: https://www.ncbi.nlm.nih.gov/geo/query/acc.cgi?acc=GSE94508). Its gene is located at chr1: 14057494–14068652, and its associated-gene symbol is PRDM2 (PR/SET Domain 2). Via Arraystar's home-made miRNA target prediction software based on TargetScan and miRanda [16], we found that hsa_circ_0005986 has hsa-miR-129-5p seed matches. And through checking the miRTarBase (http://mirtarbase.mbc.nctu.edu.tw/php/detail.php?mirtid=MIRT005412), we found *Notch1* is one of validated target genes of hsa-miR-129-5p by luciferase reporter assay and microarray [17]. Several reports have shown that *Notch1* plays an important role in HCC carcinogenesis and metastasis [18–21]. Hence, it could conceivably be hypothesized that hsa_circ_0005986 and *Notch1*mRNA may act as a pair of ceRNAs that are linked by miR-129-5p.

In this study, we first investigated the hsa_circ_0005986 expression characteristics in HCC tissues and cell lines, and explored the association between the hsa_circ_0005986 expression level and clinicopathological characteristics of patients with HCC. We then investigated whether hsa_circ_0005986 can act as a ceRNA for *Notch1* through miR-129-5p. We found that hsa_circ_0005986 could directly interact with miR-129-5p through luciferase reporter assay, and regulate *NOTCH1* mRNA expression by acting as a sponge for miR-129-5p. Finally, we knocked down the hsa_circ_0005986 expression level by using small interfering RNA (siRNA) and found that hsa_circ_0005986 affected not only cell cycle, but also cell proliferation by regulating the G_0_/G_1_ to S phase transition.

## RESULTS

### General characteristics of the subjects

There was a male dominance in studied patients. More than three-quarters (87.7 %) of patients suffering from HCC were men. The average age of HCC patients was 57.2 years. AFP negative patients accounted for a bit less than half (46.8%; Table 1).

**Table 1:** The relationship between hsa_circ_0005986 expression levels (Δ*C*t) in cancer tissues and clinicopathological factors of patients with HCC

Characteristics	No. of patients	Percent of patients (%)	Mean ± SD	*P* value
Age (years)				0.631
≥ 50	62	76.5	11.27 ± 1.40	
< 50	19	23.5	11.15 ± 0.85	
Gender				0.936
Male	71	87.7	11.25 ± 1.32	
Female	10	12.3	11.16 ± 1.09	
Family history				0.001
Positive	33	40.7	11.84 ± 1.27	
Negative	48	59.3	10.83 ± 1.14	
Diabetes Mellitus				0.156
Yes	13	14.5	10.78 ± 1.55	
No	68	85.5	11.33 ± 1.23	
Diameter (cm)				< 0.001
≥ 5	35	16	11.80 ± 1.12	
< 5	46	84	10.82 ± 1.26	
Differentiation				0.228
Well	16	19.8	11.24 ± 1.14	
Moderate	52	64.2	11.38 ± 1.38	
Poor	13	16	10.69 ± 0.95	
Microvascular invasion				0.026
Positive	25	31.2	11.70 ± 1.32	
Negative	55	68.8	11.01 ± 1.23	
Cirrhosis				0.763
Yes	48	60	11.18 ± 1.33	
No	32	40	11.26 ± 1.18	
BCLC stage				< 0.001
A	32	39.5	10.55 ± 0.98	
B + C	49	60.5	11.69 ± 1.27	
TNM stage				0.166
I	41	50.6	11.03 ± 1.20	
II	18	22.2	11.72 ± 1.49	
III + IV	22	27.2	11.24 ± 1.29	
AFP				0.690
> 20	42	53.2	11.20 ± 1.36	
≤ 20	37	46.8	11.31 ± 1.24	
AKP				
> 95	43	53.1	11.36 ± 1.23	0.397
≤ 95	38	46.9	11.11 ± 1.36	
GGT				0.410
> 50	55	54.1	11.15 ± 1.31	
≤ 50	45	45.9	11.39 ± 1.27	

### Hsa_circ_0005986 was downregulated in HCC tissues and HCC cell lines

**Figure 1:**
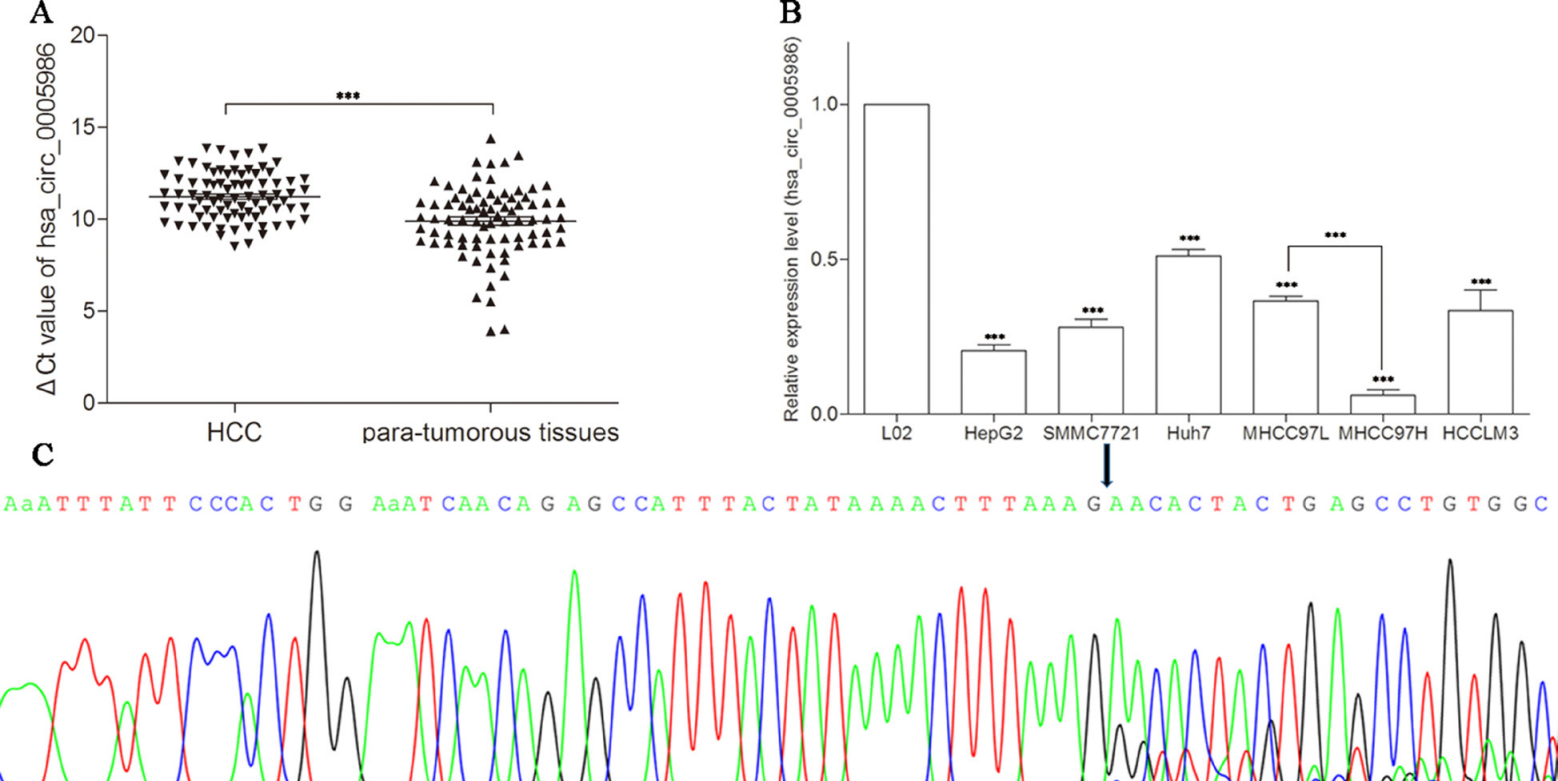
Detection of hsa_circ_0005986 expression by qRT-PCR (**A**) Decreased expression of hsa_circ_0005986 between HCC tissues and para-tumorous tissues. Data are means ± SD. (**B**) Decreased expression of hsa_circ_0005986 between HCC cell lines and human normal hepatic cell line L02. Data are means ± SD. ***P < 0.001 (**C**) Sanger sequencing showed the back-splice junction sites.

The expression of hsa_circ_0005986 in HCC tissues was significantly lower than that in matched non-tumorous tissues (*P* < 0.001) (Figure 1A), and the average relative expression level of hsa_circ_0005986 was downregulated by 2.94-fold. Furthermore, the downregulated ratio of hsa_circ_0005986 reached 80.2% (65/81).

To confirm the results of hsa_circ_0005986 in HCC tissues, we observed hsa_circ_0005986 levels in HCC cell lines (HepG2, SMMC7721, Huh7, MHCC97L, MHCC97H, and HCCLM3). The results showed that hsa_circ_0005986 levels in all detected HCC cell lines were significantly lower than those in normal hepatic cell line L02 (*P* < 0.001), and one interesting finding is the expression level in MHCC97L was significantly higher than that in MHCC97H (Figure 1B).

Sanger sequencing of the qRT-PCR products confirmed the back-splice junction sequence of hsa_circ_0005986 which was consistent with that from database (http://www.circbase.org/cgi-bin/listsearch.cgi) showed (Figure 1C).

### Correlations between hsa_circ_0005986 expression level and clinicopathological parameters

The relationship between the expression level of hsa_circ_0005986 and clinicopathological factors of patients with HCC was analyzed. Low expression of hsa_circ_0005986 was significantly associated with chronic hepatitis B family history (*P* = 0.001), tumor diameters (*P* < 0.001), microvascular invasion (*P* = 0.026), Barcelona Clinic Liver Cancer staging system (BCLC) stage (*P* < 0.001) (Table 1).

### Hsa_circ_0005986 and *NOTCH1* mRNA are targeted by miR-129-5p

**Figure 2:**
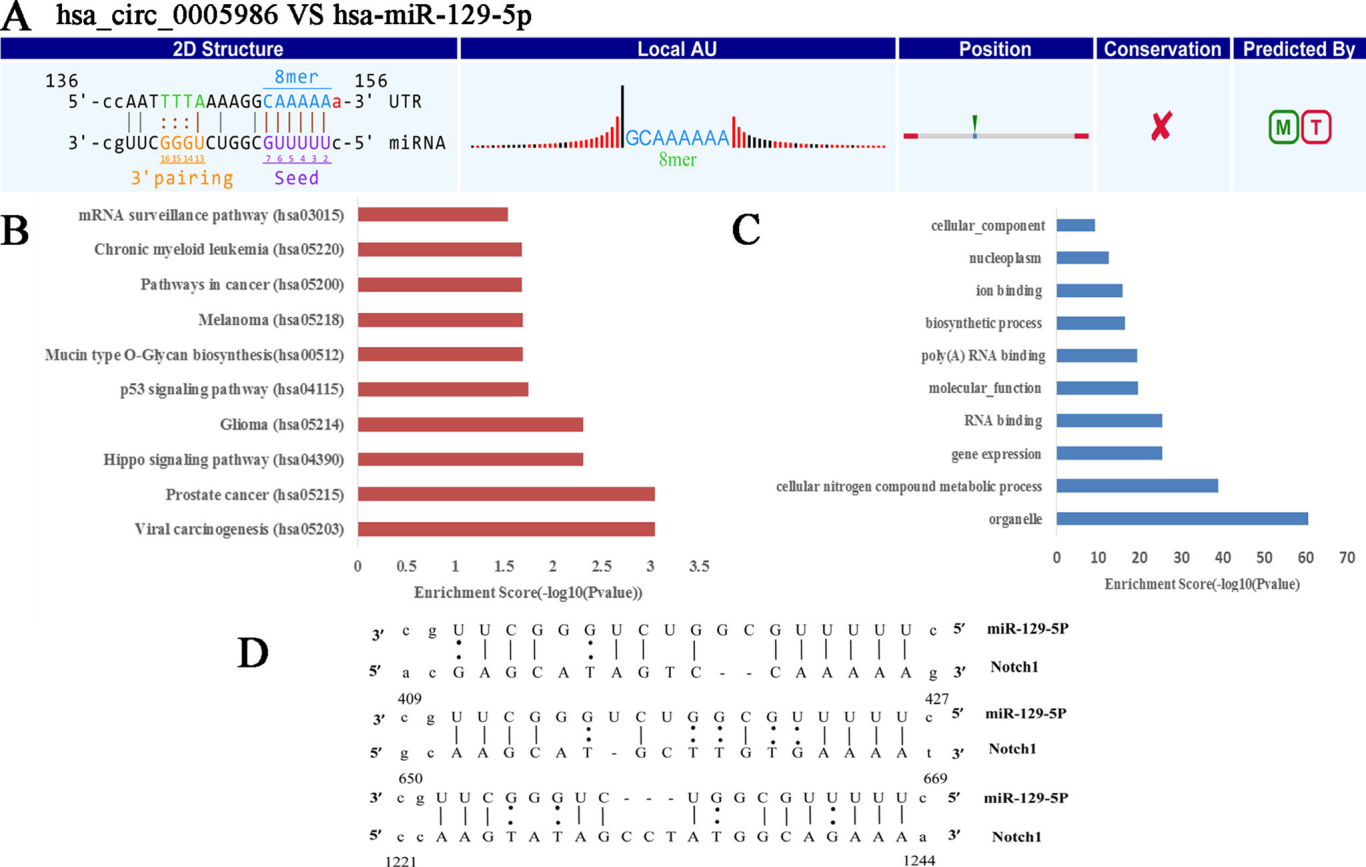
Prediction of hsa_circ_0005986-hsa-miR-129-5p-NOTCH1 interaction (**A**) The interaction of hsa_circ_0005986-hsa-miR-129-5p was predicted based on TargetScan and miRanda. (**B**) The hsa_circ_0005986-miR-129-5p related KEGG pathway analysis. (**C**) The hsa_circ_0005986-miR-129-5p related GO analysis. (**D**) *NOTCH1* is one of validated target genes of hsa-miR-129-5p (http://mirtarbase.mbc.nctu.edu.tw/php/detail.php?mirtid=MIRT005412). Detailed seed matches between hsa-miR-129-5p and *Notch1*mRNA.

**Figure 3:**
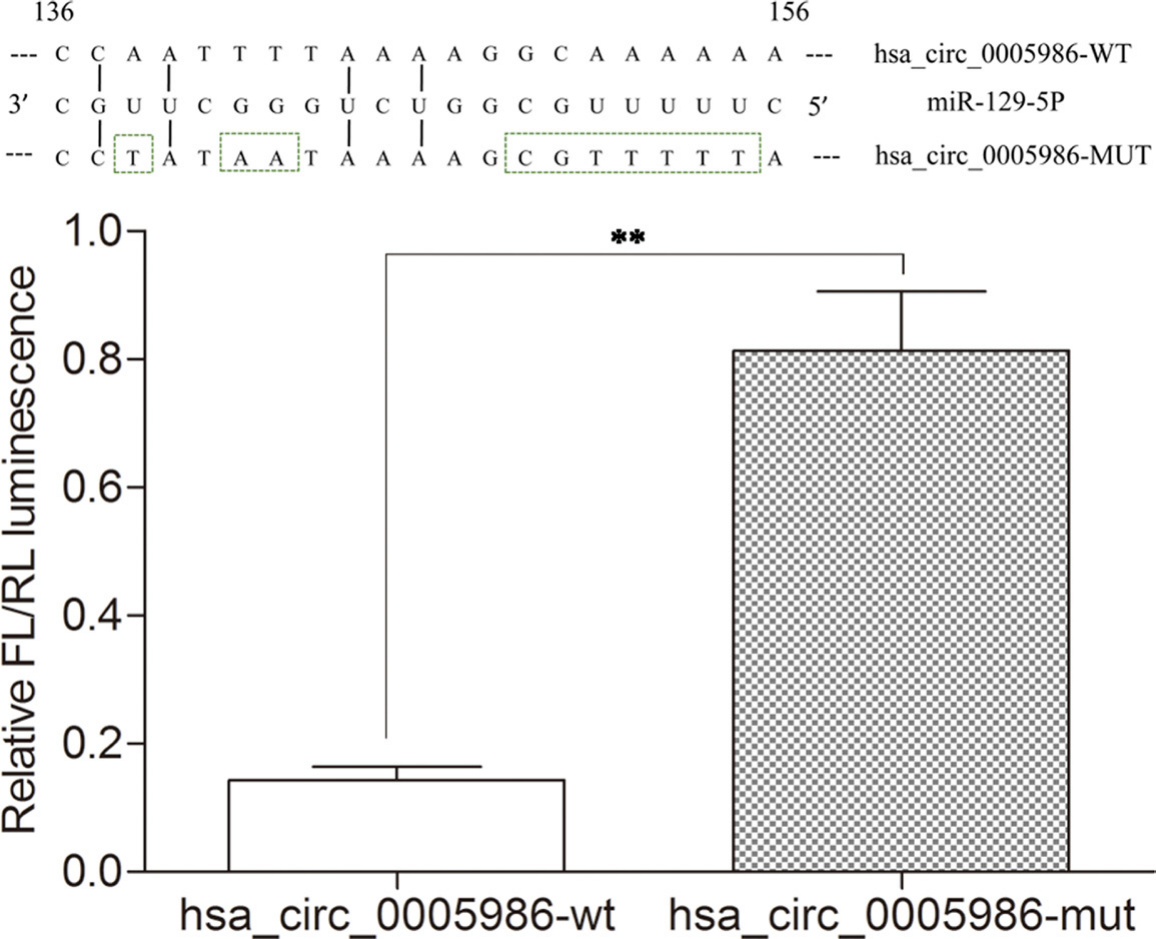
Results of dual luciferase reporter assays Relative Firefly(FL)/Renilla(RL) luminescence mediated by luciferase plasmid harboring the wild-type or mutant hsa_circ_0005986. It indicates the direct interaction between hsa_circ_0005986 and miR-129-5p. Mean ± SD, *n* = 3; **P < 0.01.

*NOTCH1* is one of the validated targets of miR-129-5p (Figure 2C) [17]. Here, our study further verified the direct interaction between hsa_circ_0005986 and miR-129-5p via dual luciferase reporter assays (Figure 3).

**Figure 4:**
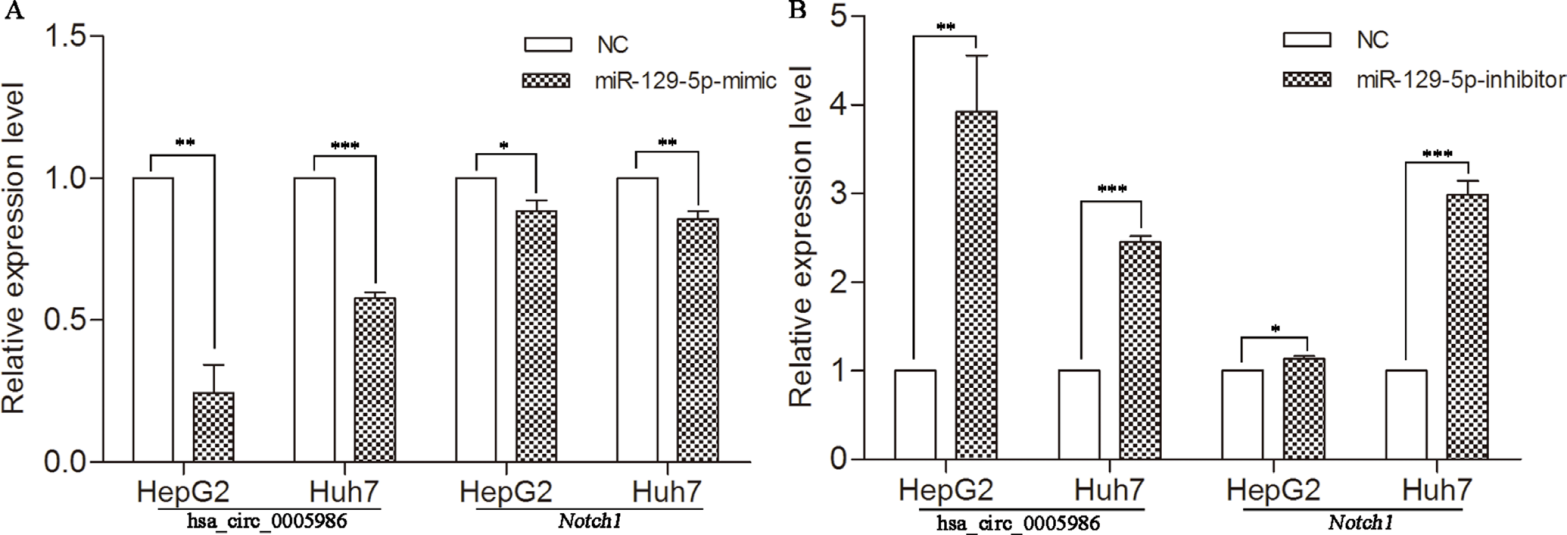
Expression of hsa_circ_0005986 and *Notch1* mRNA in HepG2 and Huh7 cell lines transfected with miR-129-5p mimics (**A**) or inhibitors (**B**). Data are presented as mean ± SD, *n* = 3. NC, negative control. *P < 0.05, **P < 0.01, ***P < 0.001.

To test whether hsa_circ_0005986 and *Notch1* expression levels were affected by miR-129-5p, we increased the miR-129-5p level by transfection of its mimics into HepG2 and Huh7 cells. qRT-PCR analysis indicated that the transfection of miR-129-5p mimics decreased not only hsa_circ_0005986 levels but also *Notch1*mRNA levels in both HepG2 and Huh7 cells (Figure 4A).

To decrease the miR-129-5p level, we transfected miR-129-5p inhibitors into HepG2 and Huh7 cells. Next, we utilized qRT-PCR analysis to reveal that miR-129-5p inhibitors increased both hsa_circ_0005986 and *Notch1* abundance in two HCC cell lines (Figure 4B).

### Effects of hsa_circ_0005986 downregulation on miR-129-5p and *Notch1* expression

**Figure 5:**
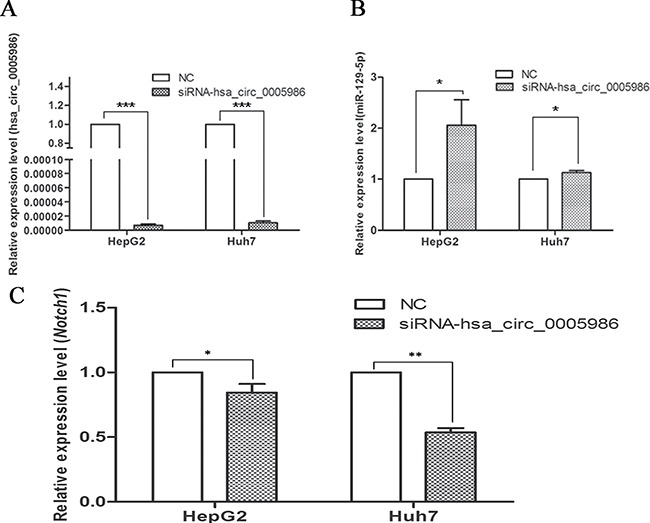
Expression of hsa_circ_0005986 (**A**), miR-129-5p (**B**) and *NOTCH1* mRNA (**C**) in HepG2 and Huh7 cell lines after hsa_circ_0005986 knockdown. Data are presented as mean ± SD, *n* = 3. NC, negative control.*P < 0.05, **P < 0.01, ***P < 0.001.

To verify that hsa_circ_0005986 and *NOTCH1* are targets of miR-129-5p, we further sought to determine whether the downregulation of hsa_circ_0005986 would influence miR-129-5p and *NOTCH1*mRNA levels. If hsa_circ_0005986 functions as a ceRNA, its downregulation might free miR-129-5p. Therefore, we designed a siRNA against hsa_circ_0005986. The results showed that this not only effectively reduced the hsa_circ_0005986 level but also increased the miR-129-5p level in the HepG2 and Huh7 cells (Figure 5A, 5B). And at the same time, the level of *NOTCH1*mRNA decreased (Figure 5C).

### Hsa_circ_0005986 regulates the cell cycle and cell proliferation

**Figure 6:**
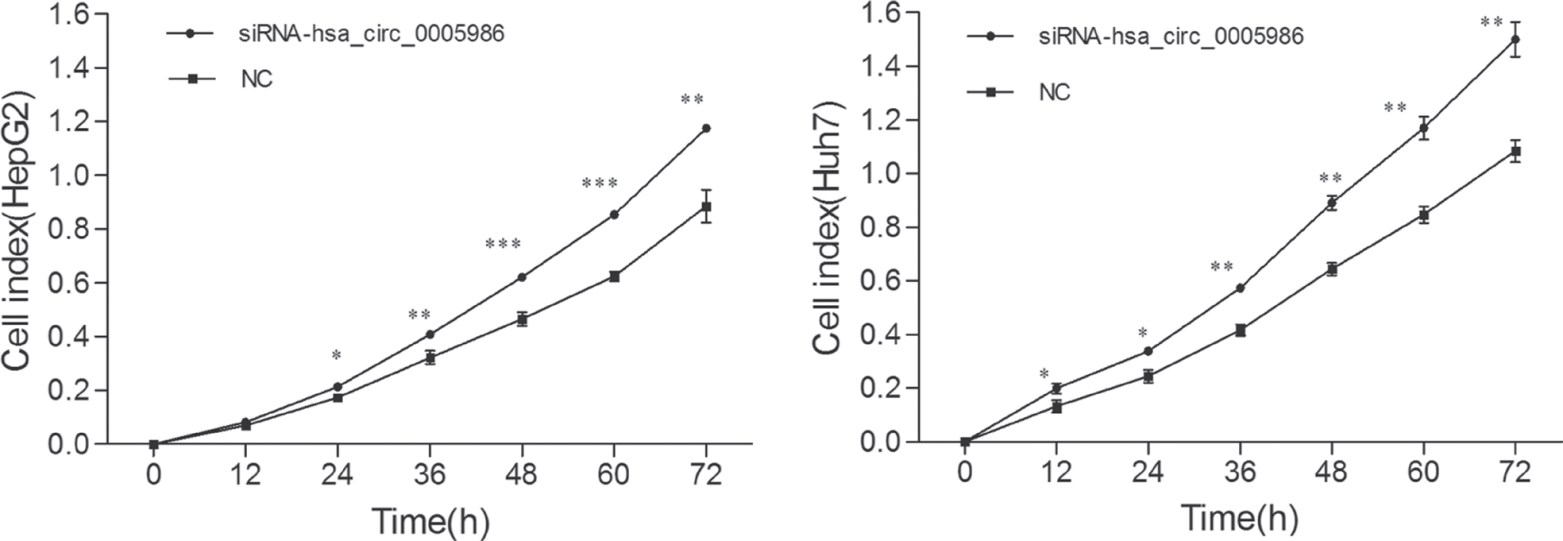
Growth curves of the HepG2 and Huh7 cell lines following by hsa_circ_0005986 knockdown The proliferation assays were performed in E-Plate 96 using a Real-Time Cell Analyzer (RTCA). Data are presented as mean ± SD, *n* = 3. NC, negative control. *P < 0.05, **P < 0.01, ***P < 0.001.

**Figure 7:**
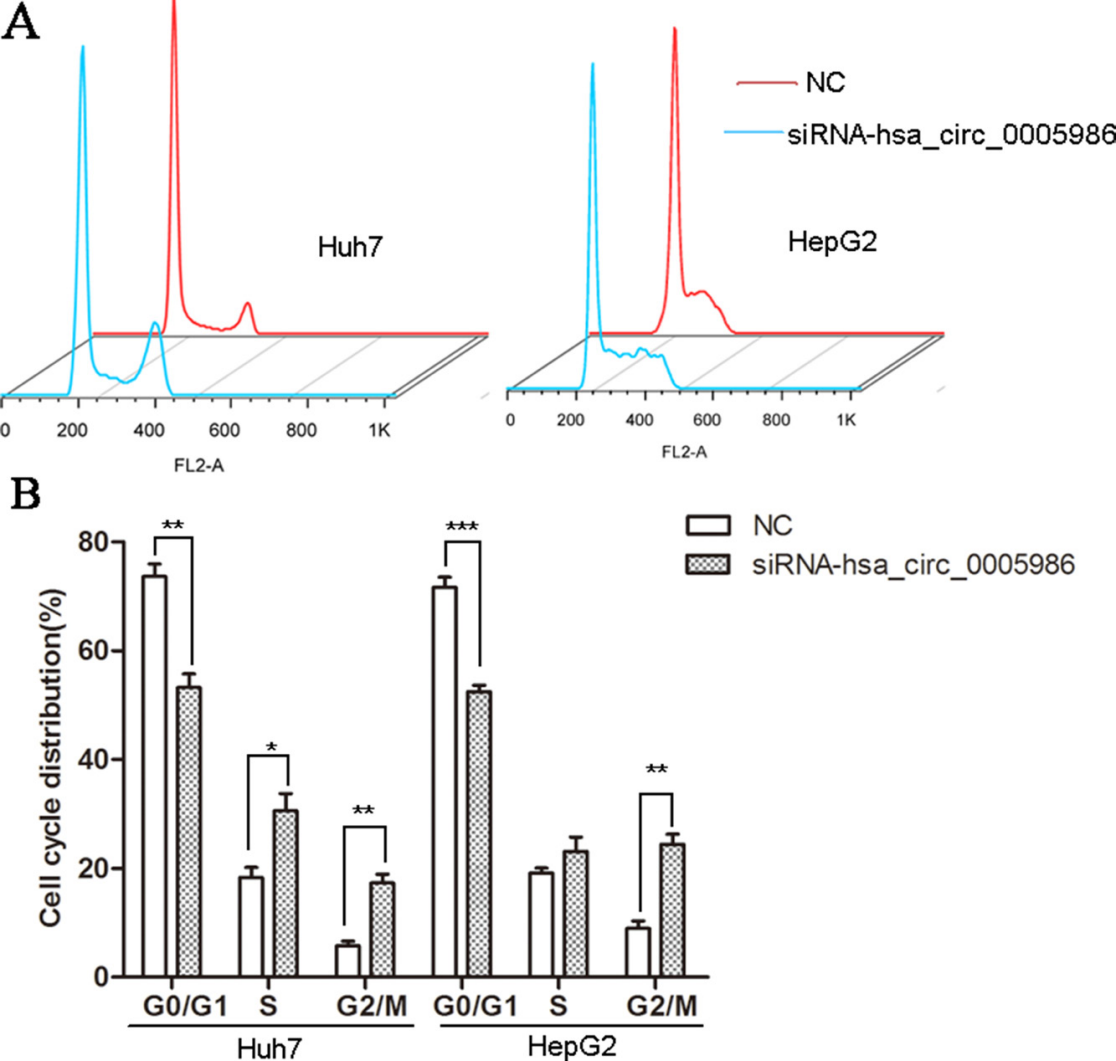
Cell cycle distributions in HepG2 and Huh7 cell lines following by hsa_circ_0005986 knockdown (**A**) Representative original flow cytometry results. (**B**) Data are presented as mean ± SD, *n* = 3. NC, negative control. *P < 0.05, **P < 0.01, ***P < 0.001.

Because perturbations of the hsa_circ_0005986 level markedly affected *NOTCH1*mRNA expression, we decided to investigate the effects of interfering hsa_circ_0005986 on the cell proliferation and cell cycle.Hsa_circ_0005986 knockdown accelerated cell proliferation in HepG2 and Huh7 cell lines (Figure 6). Flow cytometry demonstrated that hsa_circ_0005986 downregulation promoted the G_0_/G_1_ to G_2_ phase transition in HepG2 and Huh7 cells (Figure 7).

## DISCUSSION

HCC with high morbidity and mortality is one of the prevalent cancers in the world [1, 22]. The carcinogenesis of HCC has been recognized as a complex process over many years; and its clinical diagnosis often happens at an advanced stage, generally missing the best time for radical treatments. So the early diagnosis is imperative for prognosis of HCC [23]. In our study, we found that the expression level of hsa_circ_0005986 in HCC was significantly lower than that of the para-tumorous tissues (Figure 1A). More importantly, for the first time, we found that the expression level of hsa_circ_0005986 was correlated with chronic hepatitis B family history, tumor diameters, microvascular invasion, and BCLC stage (Table 1). To further observe the diagnostic value of hsa_circ_0005986 in HCC, we compared its level in HCC cell lines, and found that hsa_circ_0005986 level in HCC cell lines (noninvasive HCC cell lines HepG2 and SMMC-7721, low invasiveness cell lines Huh7 and MHCC97L, and highly invasive cell lines MHCC97H and HCCLM3) were markedly lower than those in human normal hepatic cell line L02 (Figure 1B). It is interesting to note that the expression level of hsa_circ_0005986 in MHCC97L was higher than that in MHCC97H. MHCC97L and MHCC97H are derived from a subcutaneous xenograft of metastatic human HCC in nude mice, and show different metastatic power, especially in lung [24]. Nevertheless, hsa_circ_0005986 expression level is opposite to the result of our microarray study. The reason for this is that because of the limitation of microarray sample size and HCC is one of high heterogeneous tumors, the test results have certain false positives and false negatives, so it's very important to expand the number of specimens, and use other methods (such as qRT-PCR) to verify.

Recently, circRNAs are known for being ubiquitous in cells [5, 25–26]. Thanks to improvements in high-throughput deep sequencing, circRNAs, once perceived as splicing errors, have been attracting more and more attention [7, 9, 27–28]. Sometimes, the expression levels of circRNAs can be more than ten times as high as those of their cognate linear isoforms [7, 29]. Because circRNAs are lack of the 5′cap and polyA tail, they can resist conventional RNA turnover and are more stable with a half-life more than 48 hours [25, 30, 31]. Intriguingly, scientists revealed that circRNAs can express in a complex tissue-specific, cell-type-specific or developmental-stage specific manner [9, 25, 32]. Taken together, we conclude that circRNAs could be incorporated into future clinical trials and become a kind of valuable biomarkers for precision medicine. For example, Chen et al. found that hsa_circ_0000190 harbored the diagnostic value in gastric cancer [33]. Zhang et al. disclosed that hsa_circ_101222 in combination with plasma protein endoglin strengthened the predictive value for pre-eclampsia [34].

As an enigmatic class of noncoding RNAs, circRNAs have been recently found to be associated with epigenetics and human disorders. Talhouarne et al. revealed that circular intronic RNAs can be passed on to offsprings in Xenopus oocytes and may be able to function in inheritance and epigenetics [35]. A circular isoform of antisense noncoding RNA in the INK4 locus (ANRIL) has been revealed to associate with the development of atherosclerosis [36]. *CDR1* gene transcripts (also known as circular RNA sponge for miR-7, CiRS-7) abundantly express in brain, neuroblastoma lines, renal cell carcinoma lines etc., and influence many diseases including diabetes, prion disorders and cancers [37–38]. Du et al. demonstrated that the circular RNA circ-Foxo3 is associated with cell cycle progression through forming circ-Foxo3-p21-CDK2 ternary complex [39].

The study of ceRNA represents a new approach to examining complex post-transcriptional regulatory networks. ceRNA disturbances may be linked with many diseases. ceRNAs can not only be used to explain the biological mechanism of diseases, but can also be used in drug research [40]. As an indispensable member of ceRNA network, circRNAs have been paid more and more attention to by scientists [13–15, 41–47]. For example, cir-ITCH could act as sponge of miRNAs to enhance ITCH expression and thus suppress the activation of Wnt/β-catenin signaling in ESCC, lung cancer and colorectal cancer [14, 43–44]. In human HepG2 liver cells, Caiment et al. found that O6-methylguanine DNA methyltransferase (MGMT), one of DNA damage response enzymes, could interact with miR-181a-1-3p and a portion of circRNAs, and play roles in benzo[a]pyrene-induced carcinogenesis [11]. Through DIANA mirPath v.3, we identified has-miR-129-5p was closely related with a lot of cancer-related pathways, such as viral carcinogenesis, prostate cancer, hippo signaling pathway, p53 signaling pathway, pathways in cancer, PI3K-Akt signaling pathway, etc. (Figure 2B), and was involved with a lot of biological function processes such as organelle, cellular nitrogen compound, metabolic process, gene expression, etc. (Figure 2C). We focused on hsa_circ_0005986 and *NOTCH1* in this study, because both of them are targets of miR-129-5p. Several reports have shown that miR-129-5p is linked to cancer, especially HCC [45, 46]. Based on these, by using luciferase reporter assay and qRT-PCR, we found that hsa_circ_0005986 could interact with miR-129-5p directly, that interference of the hsa_circ_0005986 level could influence miR-129-5p and *Notch1* mRNA expression levels, and that hsa_circ_0005986 and *Notch1* mRNA expression changed simultaneously. These all showed hsa_circ_0005986 could regulate *NOTCH1* expression by acting as a sponge for miR-129-5p. Finally, we knocked down hsa_circ_0005986 expression by using siRNA and found that hsa_circ_0005986 affected cell proliferation by regulating the G_0_/G_1_ to S phase transition.

In summary, as one of circRNAs, hsa_circ_0005986 was lowly expressed in HCC. Its expression level was associated with chronic hepatitis B family history, tumor size, microvascular invasion and BCLC stage. One of the mechanisms underlying hsa_circ_0005986 influencing HCC carcinogenesis is that it regulated *Notch1* expression through interacting with miR-129-5p. These indicated that hsa_circ_0005986 might not only be a potential biomarker for the diagnosis of HCC but might play important roles in carcinogenesis as well.

## MATERIALS AND METHODS

### Sample collection and ethics statement

Eighty-one HCC patients were enrolled in this study. All of them received surgeries at two cancer centers (Ningbo Lihuili Hospital and Ningbo No. 2 Hospital), from March 2013 to May 2016. Tissue samples were immediately soaked in RNAfixer Reagent (Bioteke, Beijing, China) and stored at −80°C till use. Histology was assessed independently by two experienced pathologists who were blinded to the clinical data.

This study was approved by the Human Research Ethics Committee from Ningbo University (IRB No.20100303). All patients signed the informed consent. Staging was determined by American Joint Committee on Cancer criteria and BCLC staging system. Patients with prior treatment of their tumor (such as trans-catheter arterial chemoembolization (TACE), ablation, radiotherapy, etc.) or with history of other solid tumors were excluded.

### Cell culture

HCC cell lines (HepG2, SMCC7721, and Huh7) and human normal hepatic cell line L02 were purchased from the Shanghai Institute of Biochemistry and Cell Biology, Chinese Academy of Sciences (Shanghai, China). MHCC97L, MHCC97H and HCCLM3 were from the Liver Cancer Institute, Fudan University (Shanghai, China). All cell lines were cultured with RPMI 1640 Medium (Life Technologies, Carlsbad, CA, USA) containing 10% fetal bovine serum in a humidified atmosphere of 5% CO_2_.

### RNA extraction

Total RNA was extracted from the frozen tissues and fresh cultured cells by using TRIzol (Invitrogen), based on the manufacturer's instructions. After that, 10μl diethylpyrocarbonate (DEPC)-treated water was added to make the total RNA fully dissolved. Concentration and purity of total RNA samples were measured by using the SmartSpec Plus spectrophotometer (Bio-Rad, Hercules, CA, USA). The ratio of *A*_260_/*A*_280_ was used to indicate the purity of total RNA. Samples with value 1.8-2.0 were used for further experiments.

### Reverse transcription

Following the manufacturer's instructions, cDNA was generated by using the GoScript Rverse Transcription (RT) System (Promega, Madison, WI). In brief, 1 μl random primer, 1 μl oligo (dT)15 primer, 4 μl GoScript 5× reaction buffer, 2 μl MgCl2, 1 μl nucleotide mix, 0.5 μl recombinant RNasin ribonuclease, 1 μl GoScript reverse transcriptase, and 2 μg total RNA, were added in the system and then incubated at 42°C for 1h. RT reaction and no-template control were run at the same time.

### Quantitative real-time PCR

Quantitative polymerase chain reaction (qPCR) was performed by using the GoTaq qPCR Master Mix (Promega, Madison, WI) on an Mx3005P real-time PCR System (Stratagene, La Jolla, CA, USA). A total of 5 μl cDNA product was used in a 25 μl reaction mixture which included 5.5μl DEPC-treated water, 1 μl upstream primer, 1 μl downstream primer, and 12.5 μl qPCR mix. Primers were designed with Primer3 (http://frodo.wi.mit.edu/), and synthesized by Sangon Biotech (Shanghai, China). Their sequences were as follows: for hsa_circ_0005986 (divergent primers) 5′-GAAACTGGCTGCGATATGTG-3′ (forward primer) and 5′-CACAGGCTCAGTAGTGTTCTTTAAA-3′ (reverse primer); for *Notch1*mRNA 5′-GAAACTGGCTG CGATATGTG-3′ (forward primer) and 5′-CACAGGCT CAGTAGTGTTCTTTAAA-3′ (reverse primer); for hsa-miR-129-5p 5′-CAACCTTACCTTTTTGCGGTC-3′ (forward primer) and 5′-TATGCTTGTTCTCGTCTCTG TGTC-3′ (reverse primer); for glyceraldehyde 3-phosphate dehydrogenase (GAPDH, reference gene1), 5′-TCGACAG TCAGCCGCATCTTCTTT-3′ (forward primer) and 5′-AC CAAATCCGTTGACTCCGACCTT-3′ (reverse primer); and for U6snRNA (reference gene2) 5′-ATTGGAA CGATACAGAGAAGATT-3′ (forward primer) and 5′-GG AACGCTTCACGAATTTG-3′ (reverse primer). Real-time PCR was done in triplicate. The amplification specific was confirmed by melting curve analysis. The data from qRT-PCR was analyzed by the Δ*C*t method. The Δ*C*t value was determined by subtracting the *C*t value of reference gene from the *C*t value of target gene. Larger Δ*C*t value indicates lower expression. Relative expression was calculated with the 2^−ΔΔ*C*t^ method. All results are expressed as the means ± SD of three independent experiments. All of assays were performed in a blinded fashion to clinical data.

### Cloning and sequencing of qRT-PCR products

Based on the manufacturer's instructions, the qRT-PCR product of hsa_circ_0005986 was first purified by using a UNIQ-10 PCR Product Purification Kit, and then cloned into the pUCm-T vector (Sangon Biotech, Shanghai, China). Finally, DNA sequencing was performed by Sangon Biotech Co., Ltd (Figure 1C).

### Liver function and serological tumor marker analysis

Liver function including albumin (ALB), aspartate transaminase (AST), alanine aminotransferase (ALT), alkaline phosphatase (AKP), Gamma Glutamyl Transferase (GGT), and total bilirubin was measured by Olympus AU 2700 automatic biochemical analyzer with original kits (Olympus, Tokyo, Japan). Alpha-fetoprotein (AFP) was measured with an Elecsys 2010 machine (Roche Diagnostics, Basel, Switzerland).

### Transient transfection

For the transfection of the miR-129-5p mimics, inhibitors and small interfering RNAs (siRNAs), HepG2 and Huh-7 cells (2 × 105) were seeded in 6-well plates. The following day, they were transfected with 120nM miR-129-5p mimics, inhibitors or siRNA using Lipofectamine 2000 Reagent (Life Technologies). The sequence of miR-129-5p mimic was 5′-CUUUUUGCGGUCUGGGCUUGC-3′ (sense) and 5′-AAGCCCAGACCGCAAAAAGUU-3′(antisense). The sequence of miR-129-5p inhibitor was GCAAGCCCAGACCGCAAAAAG. The sequences of three siRNA for hsa_circ_0005986 were 5′ - AACUUUAA AGAACACUACUGAGC-3′ (sense) and 5′ -UUAACUCG AAGCUGUCCUGTT-3′ (antisense); 5′ - ACUUUAAAGA ACACUACUGAG-3′ (sense) and 5′ - CUCAGUAGUGUU CUUUAAAGU -3′ (antisense); 5′ - AAACUUUAAAGA ACACUACUG-3′ (sense) and 5′ - CAGUAGUGUUCUUU AAAGUUU-3′ (antisense), and we selected 5′ - AACUUU AAAGAACACUACUGAGC-3′ (sense) and 5′ -UUAACU CGAAGCUGUCCUGTT-3′ (antisense) as the best one. The sequences of the negative control siRNAs were 5′ - AACUUUAAAGAACACUACUGAGC-3′ (sense) and 5′ - GCUCAGUAGUGUUCUUUAAAGUU-3′ (antisense). These sequences were synthesized by GenePharma Co., Ltd. (Shanghai, China).

### Dual luciferase reporter assay

MiR-129-5p expression plasmid (pmirGLO) was purchased from GenePharma Co., Ltd. (Shanghai, China). The wild-type and mutant DNA sequences were synthesized by GenePharma Co., Ltd. and cloned into pmirGLO Firefly luciferase plasmid (GenePharma). The miR-129-5p MRE of hsa_circ_0005986 wild-type and mutant sequence showed in Figure 3 following Arraystar's home-made miRNA target prediction (Figure 2A). HepG2 of 80% confluence in 24-well plates was transfected using Lipofectamine 2000 Reagent (Life Technologies) according to the manufacturer's protocol. Firefly luciferase (FL) plasmid and miR-129-5p expression plasmid were cotransfected with pRL-TK Renilla luciferase (RL) vector (Promega, Madison, WI) for normalization. After 48 h, luciferase activity was measured using Dual-Glo Luciferase Assay System (Promega). For comparison, the FL activity was normalized with RL activity. All experiments were performed in triplicate.

### Cell cycle analysis

The HepG2 and Huh7 cells were washed in PBS and fixed in 75% ice-cold ethanol at −20 °C overnight. After rehydrating with ice-cold PBS, the cells were stained with PI/RNase Staining Buffer (BD Biosciences, San Jose, CA,) and analyzed by flow cytometry on a FACSCalibur Flow Cytometer (BD Biosciences) using CellQuest Pro software.

### Cell proliferation assays

The proliferation assays were performed in E-Plate 96 using a Real-Time Cell Analyzer (RTCA) (ACEA Biosciences, San Diego, CA) according to the manufacturer's protocol.

### Prediction for hsa_circ_0005986-miR-129-5p related pathways and GO analysis

The hsa_circ_0005986-miR-129-5p related pathway and gene ontology (GO) analysis were carried out based on DIANA-miRPath V.3 [48]. All of miRNA gene targets are experimentally validated (derived from TarBase7.0) [49]. *P* < 0.05 was used as the criterion for statistical significance.

### Statistical analysis

Statistical analysis was performed according to the Statistical Product and Service Solutions (SPSS) 16.0 software package (IBM, Chicago, IL). All the graphs were plotted using GraphPad Prism 6.0 (GraphPad Software, La Jolla, CA). The data are presented as the means ± SD. Paired *t* test, independent-sample *t* test, one way analysis of variance (ANOVA), and two-tailed Student's *t*-tests were flexibly used according to actual conditions. *P* value of 0.05 or less was considered statistically significant.
